# The Entropy Complexity of an Asymmetric Dual-Channel Supply Chain with Probabilistic Selling

**DOI:** 10.3390/e20070543

**Published:** 2018-07-23

**Authors:** Yimin Huang, Qiuxiang Li

**Affiliations:** 1 School of Management & Economics, North China University of Water Resources and Electric Power, Zhengzhou 450046, China; 2 Institute of Management Science and Engineering, Henan University, Kaifeng 475001, China

**Keywords:** probabilistic selling, entropy complexity, game theory, N-S bifurcation

## Abstract

Considering consumers’ attitudes to risks for probabilistic products and probabilistic selling, this paper develops a dynamic Stackelberg game model of the supply chain considering the asymmetric dual-channel structure. Based on entropy theory and dynamic theory, we analyze and simulate the influences of decision variables and parameters on the stability and entropy of asymmetric dual-channel supply chain systems using bifurcation, entropy diagram, the parameter plot basin, attractor, etc. The results show that decision variables and parameters have great impacts on the stability of asymmetric dual-channel supply chains; the supply chain system will enter chaos through flip bifurcation or Neimark–Sacker bifurcation with the increase of the system entropy, and thus the system is more complex and falls into a chaotic state, with its entropy increased. The stability of the system can become robust with the increase of the probability that product a becomes a probabilistic product, and it weakens with the increase of the risk preference of customers for probabilistic products and the relative bargaining power of the retailer. A manufacturer using the direct selling channel may obtain greater profit than one using traditional selling channels. Using the method of parameter adjustment and feedback control, the entropy of the supply chain system will decline, and the supply chain system will fall into a stable state. Therefore, in the actual market of probabilistic selling, the manufacturers and retailers should pay attention to the parameters and adjustment speed of prices and ensure the stability of the game process and the orderliness of the dual-channel supply chain.

## 1. Introduction

The rapid development of e-commerce has created a more convenient and efficient shopping environment, shortened the sales distance between manufacturers and consumers, and enriched the sales model. In 2008, Fay and Xie put forward the concepts of probabilistic products and probabilistic selling (PS), which have attracted great attention in recent years [1]. The probabilistic product is not a specific product, but a virtual product created by a seller through a series of product sales. Probabilistic selling can expand the scope of the market and reduce the uncertainty of customer demand [2,3]. Fay and Xie [4] considered the heterogeneity of customer demand, which could be separated by means of a variety of purchase options or sales plans, and developed a formal model to explore the differences via two different mechanisms. Using opaque selling mechanisms, Anderson and Xie [5] developed an analytic model to analyze product pricing and the evolution of market segmentation. Xiao and Chen [6] considered an online retailer who sells two similar products (*A* and *B*) and probabilistic products (*A* or *B*), and modeled the problem using continuous time, discrete state, and finite horizon dynamic programming considering the choice behavior of potential customers. Fay et al. [7] considered how probabilistic selling impacts the type and number of products that a retailer should carry and found that adopting PS could alter the optimal number of products. Dan et al. [8] compared markdown selling with probabilistic selling and found that probabilistic selling could improve margin management and inventory utilization by reducing the magnitude of discounts and the amount of excess inventory. Wang et al. [9] studied the impacts of return policy on the effectiveness of the probabilistic selling model. Yang et al. [10] developed a dynamic evolution game model using the Hotelling model and analyzed the strategies of duopoly retailers with probabilistic selling or traditional selling. Zhang et al. [11] constructed a new newsboy-type inventory model and analyzed the effect of probabilistic selling on inventory decisions and the expected profit through demand reshaping and demand substitution. Li and Ma [12] developed a dynamic non-cooperative price Stackelberg game model based on incomplete information of the market, and the results showed that the increase in customers’ risk aversion can increase the stability domain of the system; the opposite is true with an increase in price discount. Zhang et al. [13] used the inventory substitution strategy and probabilistic selling strategy to solve demand uncertainty, and the results showed that the probabilistic selling strategy is more profitable when the product similarity is relatively low and the number of price-sensitive customers is high. Syam et al. [14] demonstrated the situations in which a firm can benefit or lose-out from higher sales uncertainty compared with findings from the standard principal-agent models. Giri et al. [15] developed a centralised model and decentralised model with uncertain demand and found that buyback contracts failed to coordinate such a supply chain.

The above literature studied and complemented the extant research on probabilistic selling or opaque selling in different contexts. However, they did not study probabilistic selling or opaque selling in a dual-channel supply chain environment. 

Literature on the price decisions of dual-channel supply chains, as well as decision makers’ behavior, have been studied by many scholars. Chen et al. [16] developed a Stackelberg game model of dual-channel supply chains considering the channels’ environmental sustainability and showed that the influence of the level of channel environmental sustainability on pricing decisions are different in the centralized and decentralized models. Considering the efficacy of different supply chain structures and two types of channel pricing forms, Wang et al. [17] established four game models of complementary products of a dual-channel supply chain and investigated its pricing and service decisions. Aimed at the inventory competition of perishable products, Li et al. [18] examined the pricing strategy of a dual-channel green supply chain in both centralized and decentralized cases and proposed a contract to coordinate the decentralized dual-channel green supply chain. Chen [19] developed a dual-channel supply chain model and evaluated the impact of price schemes and cooperative advertising mechanisms on dual-channel supply chain competition. Considering the regulation of mandatory carbon emission capacity, Xu et al. [20] developed the coordination contract of a dual-channel supply chain in which market demand was affected by the selling prices in both online and offline channels. Li et al. [21] developed and discussed a Stackelberg game model of a supply chain considering customer returns and pricing strategies. Zhou et al. [22] studied the influence of supply disruption risk on sales prices under the direct retailer channel and traditional retailer channel. When the dual channel supply chain uses differential and non-differential pricing scenarios, respectively, Zhou et al. [23] investigated how free riding affected the pricing or service strategies and profits of the manufacturer and traditional retailer. Zhang and Wang [24] investigated the influence of service value on the pricing strategies of a dual-channel supply chain and analyzed the evolution of the system under a long-term price forecasting mechanism. 

These works studied the price decisions as well as decision makers’ behavior in dual-channel supply chain environments and analyzed the optimal solutions of dual-channel supply chain models in different conditions. However, they did not consider the effects of customers’ risk attitude on the systems’ pricing decisions. In this paper, we will study the effects of probabilistic product pricing on decision making and system stability, considering customers’ risk attitudes for probabilistic products.

Many scholars have studied the economic system and society system and found that there are both chaotic and hyper chaotic behaviors [25,26,27]. The entropy of systems can characterize the instability degree of the system; many scholars have studied the entropy characteristics of supply chain management and the economy using the entropy theory and non-linear theory. Czyż and Hauke [28] proposed that entropy was a significant tool for the analysis of regional analysis. Dai et al. [29] analyzed the influence of parameters on the stability of a continuous dual-channel closed-loop supply chain model using entropy theory and nonlinear dynamic theory considering delayed decisions and government intervention. Lou et al. [30] analyzed the entropy complexity of a dual-channel supply chain which consisted of one manufacturer and two retailers; the manufacturer took sales as a decision-making variable in order to gain a larger market share in the competition game. Lukáš and Hofman [31] used the classical Shannon entropy approach to discuss the operational complexity of company supplier–customer relations. Zhou et al. [32] investigated the properties of six kinds of entropy based on risk measures and discovered that none of the risk measures satisfied the system’s properties. Based on the 1987 and 2008 financial crises, Gençay and Gradojevic [33] provided a comparative analysis of stock market dynamics and successfuly predicted that aggregate market expectations use risk management measures based on entropy. Considering that large consumers needed to decide their energy procurement strategy, Gao et al. [34] developed an electric energy procurement decision-making model and analyzed the entropy characteristic of the model. Zou et al. [35] proposed new effective wavelet entropy to analyze crude oil price dynamics. In this paper, considering that consumers have heterogeneity and risk aversion for probabilistic products, our primary aim is to develop a non-cooperative dynamic price Stackelberg game model considering probabilistic selling under an asymmetric dual-channel structure. Using entropy theory and dynamic theory, we analyze the complexity entropy of the asymmetric dual-channel supply chain and the effects of variables and parameters on the stability of the system through numerical simulation. The main innovations of this paper are as follows:

(1) This paper enriches the research of the asymmetric dual-channel probabilistic selling supply chain system and provides a new perspective for dual-channel research and a reference for decision-making in probabilistic selling enterprises, because policymakers are eager to formulate pricing strategies for probabilistic selling to solve the practical problems of enterprises;
(2) This paper considers the risk aversion of consumers for probabilistic products which further enriches the research on the behavior supply chain;
(3) This paper analyzes the entropy complexity and characteristics of the asymmetric dual-channel supply chain and shows that decision variables and parameters have great influence on the stability of the dual-channel supply chain, and that the supply chain system will enter chaos through flip bifurcation or Neimark–Sacker bifurcation with an increase of the system entropy, and thus the system is more complex and falls into a chaotic state with its entropy increased;
(4) The entropy analysis is applied to the pricing of probabilistic products, and the influence of parameter variations on the entropy change of an asymmetric dual channel supply chain is obtained.

The remainder of this paper is organized as follows: the problem description and model construction are given in Section 2. Section 3 focuses on the stability analysis of the Nash equilibrium point. The complexity entropy and dynamic characteristics of the dynamic probabilistic selling game model are analyzed with parameter changes in Section 4. The method of parameter adjustment and feedback control is applied to cause the probabilistic selling supply chain to go back to the stable state in Section 5. Finally, the conclusions of this paper are given in Section 6.

## 2. Model Construction

### 2.1. Model Description and Hypothesis

**Figure 1:**
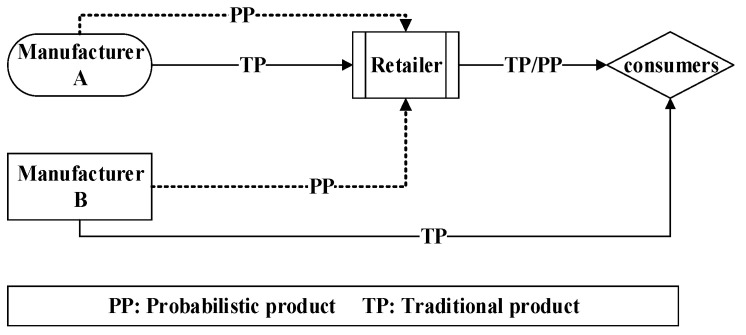
The asymmetric dual-channel supply chain system with probabilistic selling.

In this paper, we consider an asymmetric dual-channel supply chain which includes two manufacturers (A and B) and a retailer as shown in Figure 1. The two manufacturers provide two substitute products (a and b) and agree to create probabilistic products and sell them to customers through the retailer; manufacturer A sells his traditional products to customers through the retailer, while manufacturer B builds a direct channel to sell his traditional products.

The following assumptions are made to simplify our model in this paper:

(1) The two manufacturers are the leaders and the retailer is a follower, which implies that the retailer makes decisions based on the two manufacturers’ decisions;
(2) Facing the heterogeneous consumers, manufacturer A authorizes the retailer to sell the traditional product and probabilistic product and manufacturer B retains its traditional products in its direct channel and sells the probabilistic product through the retailer;
(3) In order to simplify the model, and without losing its generality, we assume the operating cost and selling cost of the retailer are normalized to zero. The production costs for the two manufacturers are normalized to zero;
(4) The demand function of heterogeneous consumers follows Hotelling distribution: one consumer can only buy one product out of the traditional product and probabilistic product; the consumer heterogeneity is expressed by an ideal point x which lies in [0, 1];
(5) The manufacturer A is located at 0 and manufacturer B is located at 1.

The main symbols used in this paper and its meanings are shown in the Table 1.

### 2.2. Model Construction

The goal of the customer is to maximize the utility of the purchasing product, and the utility of the customer from purchasing traditional product (a or b) is $v-cx-p_{a}$ or $v-c\left(1-x\right)-p_{b}$, whereas the utility of the customer from purchasing the probabilistic product is $\varphi \left(v-cx\right)+\left(1-\varphi \right)\left[v-c\left(1-x\right)\right]-p_{p}$. Considering that customers have risk aversion behavior when purchasing probabilistic products (β), $x_{a}$ and $x_{b}$ are determined by the following equations:

(1) $$\;\begin{array}{l} v-cx_{a}-p_{a}=\beta \left[\varphi \left(v-cx_{a}\right)+\left(1-\varphi \right)\left(v-c+cx_{a}\right)-p_{p}\right]\\ v-c\left(1-x_{b}\right)-p_{b}=\beta \left[\varphi \left(v-cx_{b}\right)+\left(1-\varphi \right)\left(v-c+cx_{b}\right)-p_{p}\right] \end{array}$$

The demand functions of the two manufacturers can be obtained as follows:

(2) $$\begin{array}{l} x_{a}=\frac{v+c\beta -v\beta -c\beta \varphi -p_{a}+\beta p_{p}}{c+c\beta -2c\beta \varphi }\\ x_{b}=\frac{c-v-c\beta +v\beta +c\beta \varphi +p_{b}-\beta p_{p}}{c-c\beta +2c\beta \varphi } \end{array}$$

From Equation (2), we can see that $x_{a}$ and $x_{b}$ are related to the selling price of the traditional product and probabilistic product, the risk aversion level of consumers and the probability of product a being the probabilistic product.

Note that the two manufacturers are the leaders, and the retailer is a follower, which is a Stackelberg game, and the game equilibrium is called a Stackelberg equilibrium. The two manufacturers (A and B) simultaneously make a decision on $w_{a}$, $\;w_{b}$ and $\;p_{b}$ according to the market information, then the retailer determines $p_{a}$ for the traditional product a and $p_{p}$ for the probabilistic product according to the decision-making of the two manufacturers. According to $p_{a}$, $p_{b}$ and $p_{p}$, the customers decide which products to buy based on the principle of utility maximization. Therefore, the profits of the two manufacturers and the retailer are obtained.

(3) $$\;\begin{array}{l} \pi_{A}=w_{a}x_{a}+\varphi \rho w_{a}\left(x_{b}-x_{a}\right)\\ \pi_{B}=p_{b}\left(1-x_{b}\right)+\rho w_{b}\left(1-\varphi \right)\left(x_{b}-x_{a}\right)\\ \pi_{R}=x_{a}\left(p_{a}-w_{a}\right)+\varphi \left(p_{p}-\rho w_{a}\right)\left(x_{b}-x_{a}\right)+\left(1-\varphi \right)\left(p_{p}-\rho w_{b}\right)\left(x_{b}-x_{a}\right) \end{array}\;$$

Supposing $w_{a}$, $w_{b}$ and $p_{b}$ are known, substituting $x_{a}$ and $x_{b}$ into Formula (3), making a first-order partial derivative of $\pi_{R}$ for $p_{a}$ and $p_{p}$, we can obtain the marginal profits of the retailer as follows:

(4) $$\;\begin{array}{l} \frac{\partial \pi_{R}}{\partial p_{a}}=\frac{v+c\beta -v\beta -c\beta \varphi -2p_{a}+\left(1+\beta \right)p_{p}+\left(1-\rho \varphi \right)w_{a}+\left(\rho \varphi -\rho \right)w_{b}}{c+c\beta -2c\beta \varphi }\\ \begin{array}{l} \frac{\partial \pi_{R}}{\partial p_{p}}=\frac{-c+2v+c\beta -2v\beta +\left(1+\beta \right)\left[1+\beta \left(2\varphi -1\right)\right]p_{a}+\left(2\beta \varphi -\beta -1\right)p_{b}}{c\left[\beta^{2}{\left(2\varphi -1\right)}^{2}-1\right]}\\ \begin{array}{c} \;+\frac{4\beta p_{p}+\left(\beta -\beta^{2}+2\beta^{2}\varphi -2\beta \rho \varphi \right)w_{a}+2\beta \rho \left(\varphi -1\right)w_{b}}{c\left[\beta^{2}{\left(2\varphi -1\right)}^{2}-1\right]} \end{array} \end{array} \end{array}$$

By solving the equation $\frac{\partial \pi_{R}}{\partial p_{a}}=0,\;\frac{\partial \pi_{R}}{\partial p_{p}}=0$, we can obtain the retailer’s best reply functions ($p_{a}^{*}$ and $p_{p}^{*}$ see in Appendix A):

Substituting $p_{a}^{*}$ and $p_{p}^{*}$ into Formula (3), making a first-order partial derivative of $\pi_{A}$ for $w_{a}$, $\pi_{B}$ for $w_{b}$ and $p_{b}$, we can obtain the marginal profits of the two manufacturers ($\frac{\partial \pi_{A}}{\partial w_{a}}$, $\frac{\partial \pi_{B}}{\partial w_{b}}$, $\frac{\partial \pi_{B}}{\partial p_{b}}$ see Appendix A).

The optimal response functions (the Nash equilibrium point) of two manufacturers can be obtained in accordance with $\frac{\partial \pi_{A}}{\partial w_{a}}=0$, $\frac{\partial \pi_{B}}{\partial w_{b}}=0$, $\frac{\partial \pi_{B}}{\partial p_{b}}=0$. The expressions of the optimal response function of two manufacturers are very complicated; the relationship between variables and parameters cannot be seen from the expression functions. Next, we will construct a dynamic game model to study the evolution characteristics of the Nash equilibrium point.

The price decisions of the two manufacturers are not completely rational because they cannot get all the necessary information in the market, so the two manufacturers have limited rational behavior when making decisions. In this paper, we develop a dynamic Stackelberg price game model considering probabilistic selling and focus on the entropy complexity analysis and dynamic characteristics of the asymmetrical dual-channel supply chain. We assume the two manufacturers use bounded rational expectations and adopt the myopic adjustment mechanism. With an asymmetric dual-channel structure and a different channel power, the three-dimensional dynamic price game model considering probabilistic selling is as follows:

(5) $$\;\begin{array}{c} w_{a}\left(t+1\right)=w_{a}\left(t\right)+n_{1}w_{a}\left(t\right)\frac{\partial \pi_{A}\left(t\right)}{\partial w_{a}\left(t\right)}\\ w_{b}\left(t+1\right)=w_{b}\left(t\right)+n_{2}w_{b}\left(t\right)\frac{\partial \pi_{B}\left(t\right)}{\partial w_{b}\left(t\right)}\\ p_{b}\left(t+1\right)=p_{b}\left(t\right)+n_{3}p_{b}\left(t\right)\frac{\partial \pi_{B}\left(t\right)}{\partial p_{b}\left(t\right)} \end{array}\;$$

where $n_{1},\;n_{2}$ and $n_{3}$ are the price adjustment speed parameters of the two manufacturers according to their marginal profits, which reflect the manufacturer’s learning behavior and active managerial behavior. When the marginal profit in period t is greater than zero, the manufacturers will increase the prices in period t+1; on the contrary, the manufacturers will decrease the prices in period t+1.

## 3. The Stability of System (5)

In this section, we will analyze the stability of system (5). Because of the complexity of the model, the Nash equilibrium solution of the model is very complex, and we cannot see the interaction between variables and parameters. Here, we assign values to parameters according to the current situation and characteristics of the supply chain enterprises, and a data survey of enterprises and customers in Taobao—v=15, c=6, φ=0.45, ρ=0.95, β=0.8—and study the stability of the model through numerical simulation.

When $w_{a}\left(t+1\right)=w_{a}\left(t\right)$, $w_{b}\left(t+1\right)=w_{b}\left(t\right)$ and $p_{b}\left(t+1\right)=p_{b}\left(t\right)$, we can get eight equilibrium solutions for system (5):

$E_{1}\left(w_{a},\;w_{b},\;p_{b}\right)=\left(0,\;0,\;0\right)$;
$E_{2}\left(w_{a},\;w_{b},\;p_{b}\right)=\left(0,\;0,\;3.94\right)$;
$E_{3}\left(w_{a},\;w_{b},\;p_{b}\right)=\left(0,\;4.23,\;0\right)$;
$E_{4}\left(w_{a},\;w_{b},\;p_{b}\right)=\left(3.302,\;0,\;0\right)$;
$E_{5}\left(w_{a},\;w_{b},\;p_{b}\right)=\left(0,\;7.822,\;8.913\right)$;
$E_{6}\left(w_{a},\;w_{b},\;p_{b}\right)=\left(5.71,\;0,\;6.295\right)$;
$E_{7}\left(w_{a},\;w_{b},\;p_{b}\right)=\left(3.132,\;2.27,\;0\right)$;
$E_{8}\left(w_{a},\;w_{b},\;p_{b}\right)=\left(9.895,\;14.96,\;14.32\right).$

We can see that $E_{1},E_{2},\;E_{3},E_{4},\;E_{5},E_{6},E_{7}$ are the boundary equilibrium points because they are partly or entirely zero; the decision variables obviously are not allowed to be zero in economics for decision makers, so $E_{1}\;\sim \;E_{7}$ are unstable and $E_{8}$ is the only Nash equilibrium point. It is meaningless to study the unstable equilibrium solution, so we only analyze the Nash equilibrium point.

The Jacobian matrix of system (5) at the Nash equilibrium point is

(6) $$J=\left|\begin{array}{ccc} 1-1.39n_{1} & 0.09n_{1} & 0.49n_{1}\\ 0.09n_{2} & 1-0.96n_{2} & 1.23n_{2}\\ 0.54n_{3} & 1.29n_{3} & 1-2.85n_{3} \end{array}\right|$$

The characteristic equation of system (5) at the Nash equilibrium point is

(7) $$-\lambda^{3}+A\lambda^{2}+B\lambda +C=0\;$$

where

$A=-3-1.385n_{1}-0.958n_{2}-2.851n_{3}$
$B=-3+2.77n_{1}+1.916n_{2}-1.318n_{1}n_{2}+5.702n_{3}-3.689n_{1}n_{3}-1.143n_{2}n_{3}$
$C=1-1.385n_{1}-0.958n_{2}+1.318n_{1}n_{2}-2.851n_{3}+3.689n_{1}n_{3}+1.143n_{2}n_{3}-1.19n_{1}n_{2}n_{3}$

In order to guarantee that the Nash equilibrium point is locally stable, A, B and C must satisfy the following conditions:

(8) $$\{\begin{array}{l} 1+A+B+C>0\\ -1+A-B+C<0\\ \begin{array}{l} 1-C^{2}>0\\ {\left(1-C^{2}\right)}^{2}-{\left(B-AC\right)}^{2}>0 \end{array} \end{array}$$

By solving condition (8), the stability domain of system (5) can be obtained. Due to these limitations being so complex, solving the inequality of Equation (8) is very complicated. If the Nash equilibrium point satisfies the inequality of Equation (8), we may ensure that system (5) is locally stable. In the next section, we will prove the entropy complexity and the dynamic characteristics of system (5) through numerical simulation. 

## 4. The Complex Dynamics of System (5)

In this section, we will explore the entropy complexity and dynamic behavior of system (5) through the basin of attraction, a bifurcation diagram, the entropy of the system, the largest Lyapunov exponents (LLE), et al. 

### 4.1. The Stable Region of System (5) for Parameter Changes

In this section, we will use a 2D parameter bifurcation diagram (parameter plot basin), which is a more powerful tool for the numerical analysis of a dynamic system than that of the 1D bifurcation diagram, to analyze the effects of parameter changes on system stability [36]. 

**Figure 2:**
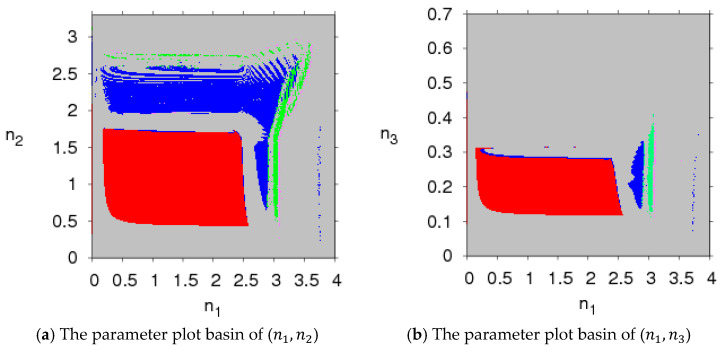
The parameter plot basin of the system (5) with respect to ($n_{1},\;n_{2}$) and ($n_{1},\;n_{3}$).

Figure 2 presents the parameter plot basin with respect to the parameter ($n_{1},\;n_{2}$) and parameter ($n_{1},\;n_{3}$) with φ=0.45, ρ=0.95, β=0.8, in which we use different colors to describe different states of system (5); for example, stable states are red, period 2 is blue, 4 is green, 8 is pink, and chaos and divergence are grey (divergence means that one of the decision makers will withdraw from the market). In the red region, system (5) will stabilize in the Nash equilibrium point after multiple iteration cycles. Once the adjustment parameter goes out of the red region, system (5) will become unstable and enters a chaotic state through period-doubling bifurcation or N-S bifurcation.

**Figure 3:**
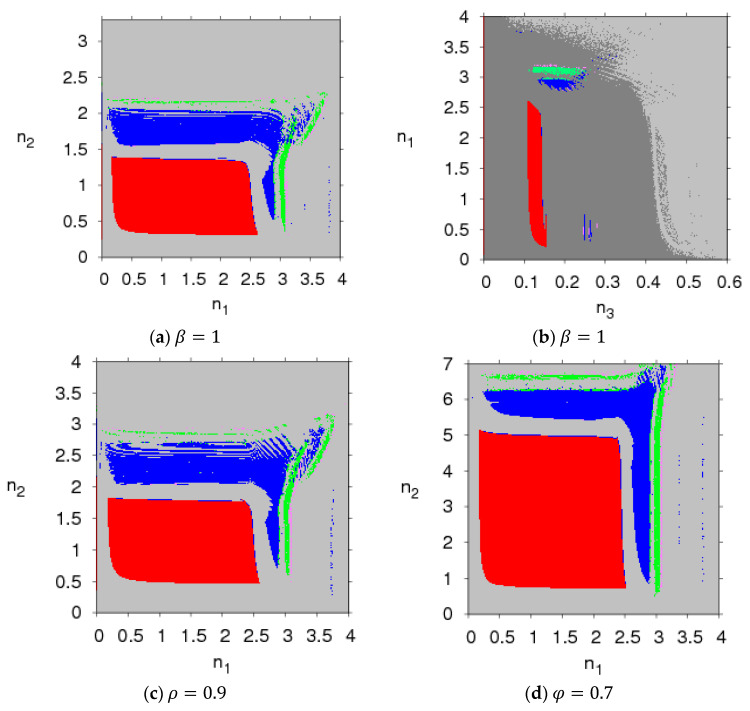
The parameter plot basin of the system (5).

Figure 3 presents the parameter plot basins of system (5) with different parameter values. Comparing Figure 3 to Figure 2, we can see that the stable regions (red region) become smaller with the increase of β and ρ and larger with the increase of φ. That is to say, the greater the consumers’ risk aversion level for purchasing probabilistic products and the relative bargaining power of the retailer are, the smaller the stable region of system (5) is; the greater the probability that product a will become a probabilistic product is, the larger the stable region of system (5) is. An increase in the stable region means more competition in system (5).

Under this unbalanced sales channel, the stability of the system can be robust with an increase of the probability of product a becoming a probabilistic product, and it weakens with the increase of the consumers’ risk aversion level for purchasing probabilistic products and the relative bargaining power of the retailer, which is different from the literature [12].

**Figure 4:**
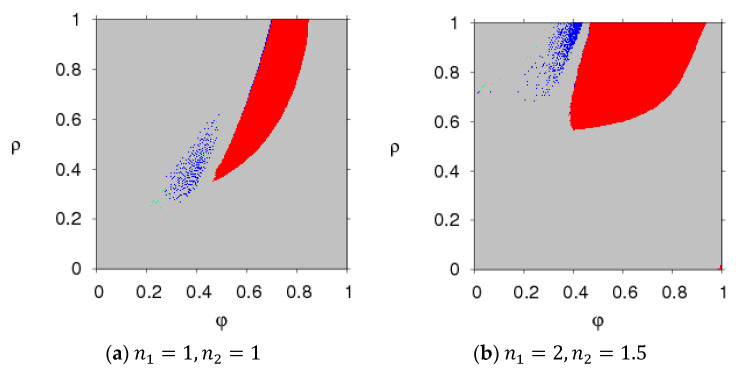
The parameter plot basin of system (5) with the parameter group (φ, ρ).

**Figure 5:**
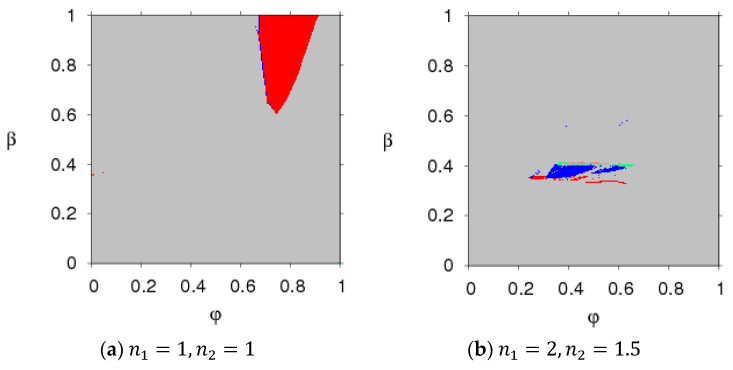
The parameter plot basin of for system (5) with the parameter group (φ, ρ) and (φ, β).

Figure 4 and Figure 5 present the parameter plot basins with respect to parameter (φ, ρ) and parameter (φ, β) when $n_{1}=1,\;n_{2}=1$ and $n_{1}=2,\;n_{2}=1.5$, respectively. We can see that when the two manufacturers keep a certain price adjustment speed, system (5) can stay in the Nash equilibrium point when the parameter group (φ, ρ) and parameter group (φ, β) are all in the red region, losing the Nash equilibrium point and going into a chaotic state from the stable state when the parameters move out of the red region. With an increase of $n_{1},\;n_{2}$, the stable regions of system (5) with parameter group (φ, β) almost vanish, and there is only a small region in which system (5) displays two-period motion.

In summary, the stability of the asymmetric dual-channel probabilistic selling supply chain is affected by the parameter values and the price adjustment speeds. When the parameter value is beyond the stable region, chaotic and periodic behaviors will appear. There are price fluctuations in the chaotic state which may result in unstable profits and lagging sales. Thus, the two manufacturers should take appropriate parameter values and price adjustment speeds to make sure that system (5) is in a stable state. 

### 4.2. The Entropy Complexity Analysis of System (5) with the Price Adjustment Speed ($n_{1}$)

**Figure 6:**
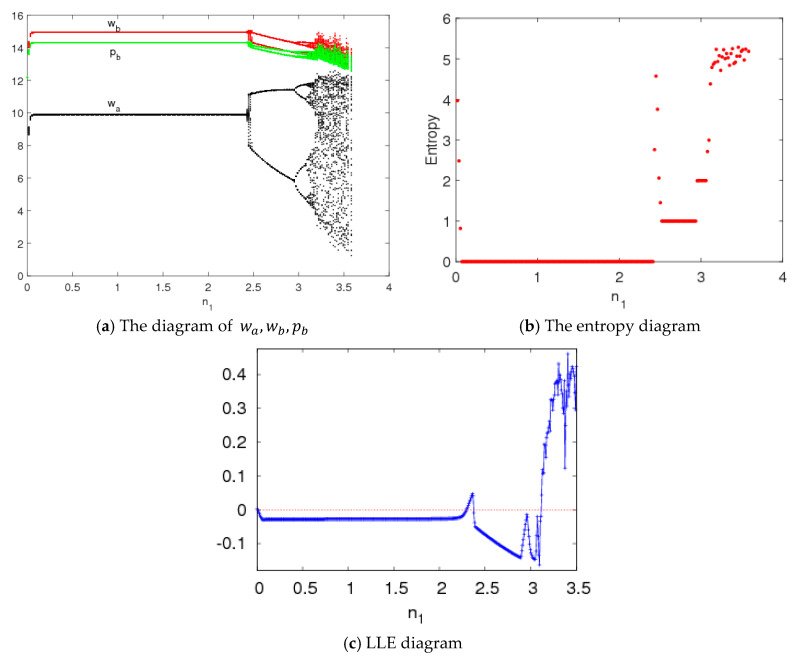
Bifurcation diagram, entropy diagram and LLE diagram of system (5) with changes of $n_{1}$ when $n_{2}=1.5,\;n_{3}=0.2$.

In this section, we also set the parameters values as v=15, c=6, φ=0.45, ρ=0.95, β=0.8. The dynamic behaviors and entropy of system (5) are described with variations of $n_{1}$. Figure 6 shows the wholesale price, entropy diagram and the LLE of system (5) as $n_{1}$ changes. We can see that system (5) is stable when $n_{1}<2.35$, and the bifurcation and chaos in system (5) occur through period-doubling bifurcation when $n_{1}$ increases. For the asymmetric sale channel in this paper, the wholesale price of manufacturer B using direct selling is larger than that of manufacturer A using indirect selling. Manufacturer B gives a lower retail price $p_{b}$ than the wholesale price $w_{b}$ to get more channel power, which conforms to the operation of the real market. 

We know that entropy can measure the chaotic degree of the system; the system entropy is small when the system is in a stable state, and the system entropy is large when the system is in a chaotic state. On the other hand, the system entropy shows the probability of the occurrence of some particular information; when the entropy of the system is high, we need more information to make the system clear. The equation of entropy used in this paper is as follows:

(9) $$S\left(p_{1},\;p_{2},\cdots ,p_{n}\right)=-\overset{n}{\underset{i=1}{\sum }}p_{i}\log_{2}p_{i}$$

There will inevitably be many uncertain factors in the complex and changeable market. In this paper, entropy theory is applied to the study of the complexity of supply chains. Through the simulation analysis, we can clearly see the effect of parameter changes on the entropy of the system of the dual-channel supply chain, and then we can quantify the stability of the supply chain system using entropy, which lays the foundation for further effectively controlling the complexity of the whole supply chain.

From Figure 6b, when $n_{1}$ is less than 2.36, the wholesale prices and retail prices are certain values, and system (5) is stable and the entropy of system (5) is equal to 0. With $n_{1}$ increasing, the wholesale prices and retail prices have multiple values, which show that system (5) is in a periodic motion state and the entropy of system (5) increases. When $n_{1}>3.15$, system (5) is in a chaotic state and the entropy of system (5) is high. Thus, we can make a conclusion that irrational changes of the price adjustment parameter will lead to a larger entropy of the system, and the two manufacturers must get more market information to rationally make price adjustments and keep the system in a stable state.

Figure 6c is LLE diagram, which can reflect the state of system (5). When LLE is negative, system (5) remains stable with lower entropy. When the majority of the LLE are positive, the dynamic system (5) falls into chaos with higher entropy. In other words, the larger the positive Lyapunov exponent is, the more chaotic the system is and the greater the entropy is. We can see from Figure 6 that the price evolution diagram corresponds to the entropy diagram and maximal Lyapunov exponent diagram.

**Figure 7:**
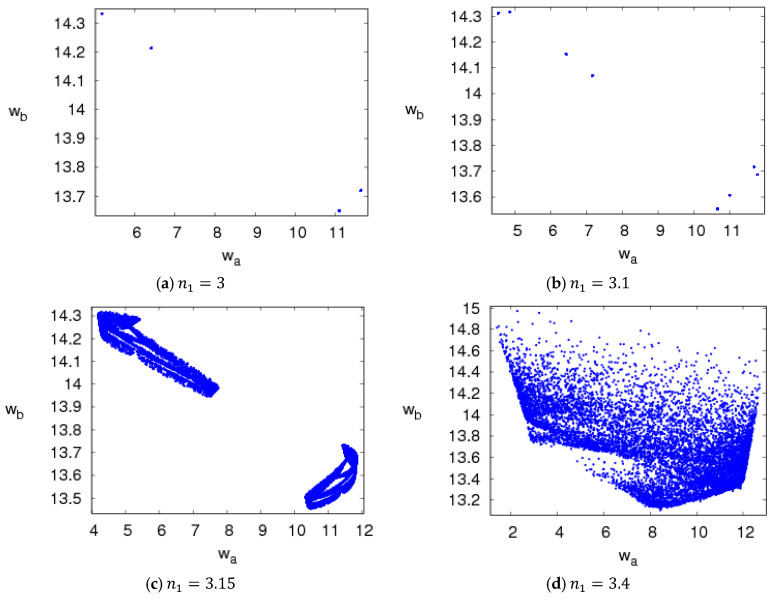
Chaos attractor of system (5) with $n_{2}=1.5,\;n_{3}=0.2$.

**Figure 8:**
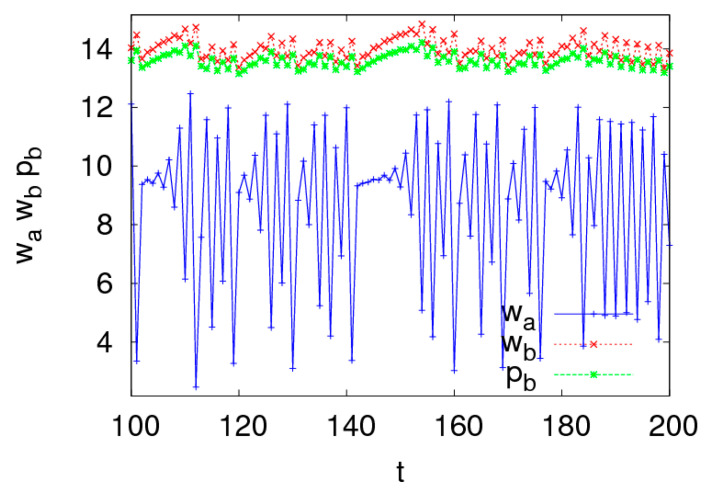
The power spectrum of system (5) when $n_{1}=3.3,\;n_{2}=1.5,\;n_{3}=0.2$.

The attractors of system (5) are shown in Figure 7. For $2.35<n_{1}\leq 3.1$, system (5) is in a period bifurcation state and its attractors are shown in Figure 7a,b; when $3.1<n_{1}\leq 3.5$, system (5) is in the chaotic states and its attractors are shown in Figure 7c,d. Figure 8 shows the power spectrum of system (5) when $n_{1}=3.3,\;n_{2}=1.5,\;n_{3}=0.2$, which is another chaotic feature of system (5).

Figure 2a shows that the evolution process of system (5) is similar when $n_{1}\;and\;n_{2}$ change, so we will not study the evolution of system (5) when $n_{2}$ is changing.

### 4.3. The Entropy and Neimark–Sacker Bifurcation of System (5) with $n_{3}$ Changing

Considering the asymmetry of the dual-channel supply chain structure in this paper, the effect of price adjustment speed ($n_{3}$) on the stability characteristics of system (5) is analyzed in this section.

**Figure 9:**
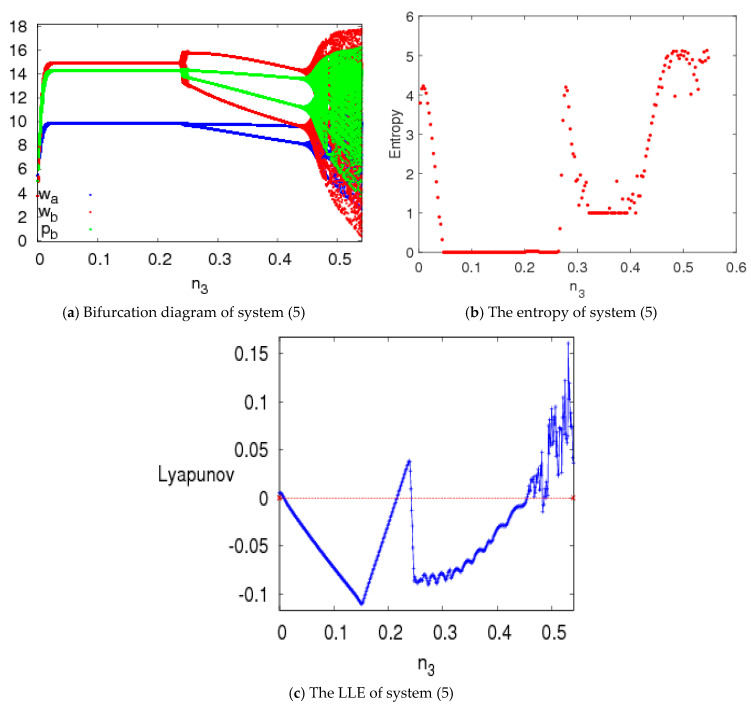
The evolution characteristics of system (5) with $n_{3}\in \left(0,\;0.55\right)$.

Figure 9 shows the dynamic characteristics of system (5) with respect to $n_{3}\in \left(0,\;0.55\right)$ and $n_{1}=1.5,\;n_{2}=1.5$ when other parameters are fixed as above. We can see that when $n_{3}$ is small, the Nash equilibrium point is locally stable, the entropy of system (5) is low and the LLE is negative; when $n_{3}$ increases, system (5) shows a two-period bifurcation and then goes into chaotic state eventually via Neimark–Sacker bifurcation; the entropy of system (5) increases when system (5) is more unstable. 

**Figure 10:**
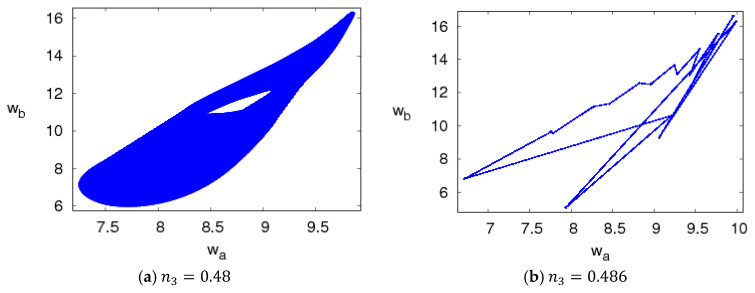
Chaos attractors of system (5) with $n_{1}=1.5,\;n_{2}=1.5$.

The chaos attractors of system (5) are simulated with different values of $n_{3}$ when other parameters are fixed and are shown in Figure 10. We can see that system (5) presents complex behaviors when $n_{3}$ increases.

Above all, we can see that the price adjustment speed ($n_{i},\;i=1,\;2,\;3$) has a great effect on the dynamics of system (5). When the price adjustment speeds are in a stable state, system (5) is stable and is in a low entropy state. When one of the price adjustment speeds increases out of the stable region, system (5) gradually goes into a chaotic state through flip bifurcation and Neimark–Sacker bifurcation and is in a high entropy state, which represents the complex dynamic behaviors.

### 4.4. The Dynamic Characteristics of System (5) with φ and ρ Changing

In this section, the dynamic characteristics of system (5) are analyzed with changes of φ and ρ when system (5) is in a stable state and chaotic state.

**Figure 11:**
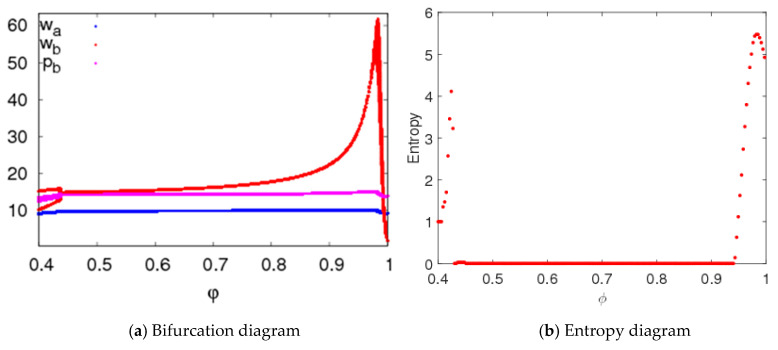
The bifurcation diagram and entropy of system (5) when φ changes.

System (5) is in a stable state when $n_{1}=1.5,n_{2}=1.5,\;n_{3}=0.2$, with other parameters fixed as above. Figure 11 shows the price bifurcation diagrams and entropy of system (5) with changes of φ. When 0.4≤φ≤0.433, system (5) is in a two-period; when 0.433≤φ≤0.94, system (5) is in a stable state; when 0.94≤φ≤1, system (5) is in a chaotic state. We can see from Figure 11b that the entropy of system (5) will increase when system (5) is in an unstable state.

**Figure 12:**
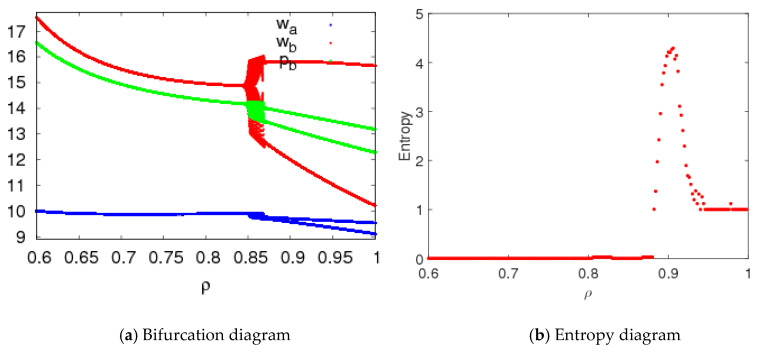
Bifurcation diagram and entropy of system (5) with ρ changing.

Figure 12 shows that the price bifurcation diagrams and entropy of system (5) with changes of ρ when $n_{1}=1.5,n_{2}=1.5,\;n_{3}=0.2$. When 0.6≤ρ≤0.88, system (5) is in a stable state, and the entropy of system (5) is close to zero; when 0.85≤ρ≤0.946, system (5) is in a chaotic state and has larger entropy; when 0.946<ρ≤1, the system (5) is in a two-period state and the entropy of system (5) is almost equal to 1.

**Figure 13:**
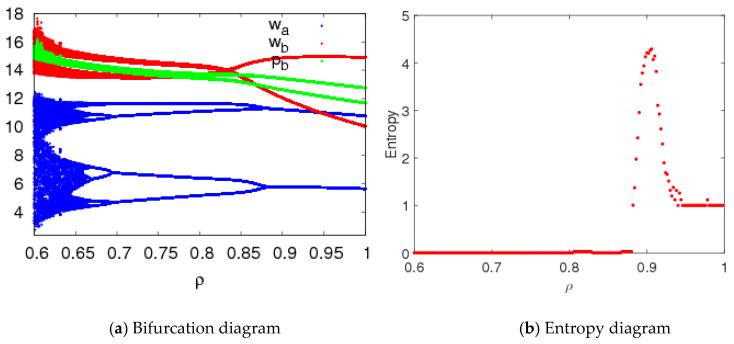
Bifurcation diagram and entropy of system (5) with ρ changing.

When $n_{1}=3,n_{2}=1.5,\;n_{3}=0.2$, the system (5) is in a chaotic state. Figure 13 gives the price bifurcation diagram and entropy of system (5) with ρ changing when $n_{1}=3,n_{2}=1.5,\;n_{3}=0.2$. We can see that system (5) can return to a period moving state from chaotic state, and the entropy of system (5) gradually reduces with an increase of ρ.

### 4.5. The Effect of Parameter Changes on Profit

**Figure 14:**
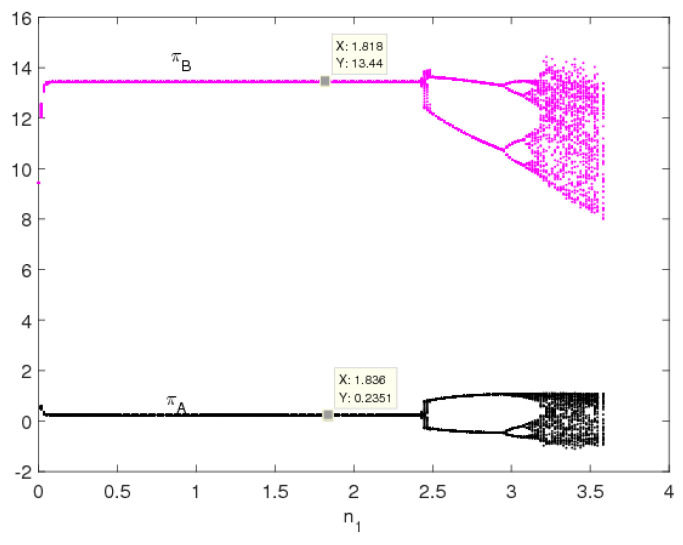
Bifurcation diagram of profits of system (5).

**Figure 15:**
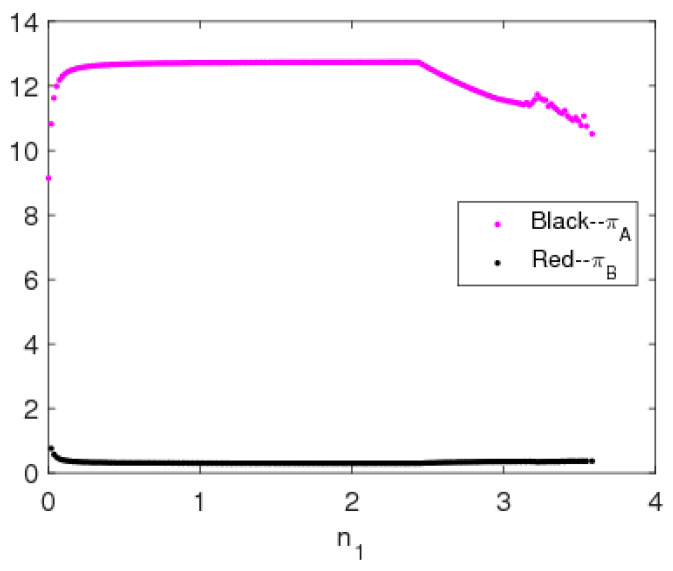
The evolutionary process of average profits of system (5).

Figure 14 is the profit bifurcation diagram of system (5). We can see that when the price adjustment speed increases, the profits of the two manufacturers become unstable and go into chaos through period-doubling bifurcation; the profit of manufacturer B is greater than that of manufacturer A, which means that the manufacturer using a direct channel can gain greater profits. Figure 15 is the evolutionary process of the average profits of system (5); we can see that the average profits of manufacturer B decrease and the average profits of manufacturer A remain unchanged in the unstable state. 

## 5. Chaos Control

All the participants want to achieve their own business objectives easily and adjust their price decisions frequently to adapt to the changes of market competition. Once the price adjustment speed losses are controlled, the market will obe ut of order and fall into chaos finally; chaos is harmful to the stability of the supply chain system. Therefore, some measures should be taken to delay or eliminate the occurrences of bifurcation and chaos.

As far as we are concerned, the parameter adjustment and feedback control method is widely applied to control the chaos of the supply chain. Xu and Ma [37] and Ma and Xie [38] had used this method to control the chaos in the insurance market and the suppy chain system. The controlled system of system (5) can be written as

(10) $$\begin{array}{c} w_{a}\left(t+1\right)=\left(1-v\right)[w_{a}\left(t\right)+n_{1}w_{a}\left(t\right)\frac{\partial \pi_{A}\left(t\right)}{\partial w_{a}\left(t\right)}]+vw_{a}\left(t\right)\\ w_{b}\left(t+1\right)=\left(1-v\right)\left[w_{b}\left(t\right)+n_{2}w_{b}\left(t\right)\frac{\partial \pi_{B}\left(t\right)}{\partial w_{b}\left(t\right)}\right]+vw_{b}\left(t\right)\\ \begin{array}{c} p_{b}\left(t+1\right)=\left(1-v\right)\left[p_{b}\left(t\right)+n_{3}p_{b}\left(t\right)\frac{\partial \pi_{B}\left(t\right)}{\partial p_{b}\left(t\right)}\right]+vp_{b}\left(t\right) \end{array} \end{array}$$

where v represents the chaos control parameter. Selecting an appropriate value for v is essential to delay bifurcation, which can make the supply chain system return to a stable state.

**Figure 16:**
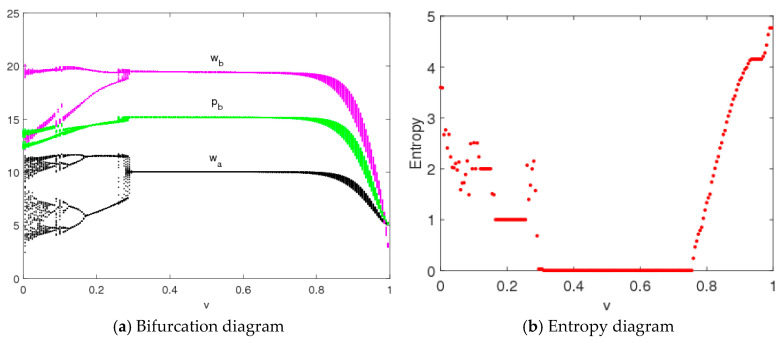
Bifurcation diagram and entropy diagram of system (9) with changes of v when $n_{1}=3,n_{2}=1.5,\;n_{3}=0.2$.

Figure 16 is the bifurcation diagrams and entropy diagram of the controlled system (9) with changes of v when $n_{1}=3,n_{2}=1.5,\;n_{3}=0.2$. The controlled system (9) is in a stable state and has low entropy when 0.3≤v≤0.86; the entropy of the control system (9) increases with an increase of system instability. Thus, we see that the controlled system (9) can return to a stable state from a chaos state with appropriate control parameter values.

## 6. Conclusions

In this paper, a dynamic Stackelberg price game model of an asymmetric dual-channel probabilistic selling supply chain is developed considering consumers’ risk aversion behavior for probabilistic products. The two manufacturers providing two substitute products all agree to create a probabilistic product and sell this to customers through the retailer; one manufacturer sells his traditional products to customers by the retailer, and another manufacturer builds a direct channel to sell his traditional products. We analyze the effects of parameter changes on the entropy and complex characteristics of the game model. The results show that decision variables and parameters should be kept in a certain range; otherwise, the system will enter chaos through flip bifurcation or Neimark–Sacker bifurcation. The system complexity is higher with the increase of entropy. The stability of the system can be robust with an increase of the probability of product a becoming a probabilistic product and will weaken with the increase of customers’ risk aversion level and the relative bargaining power of the retailer. A manufacturer using a direct selling structure may obtain greater profit than one using traditional selling channels. The method of parameter adjustment and feedback control is used to make the chaotic system return to a stable state. The research results of this paper have very important theoretical and practical values for the probabilistic selling supply chain system.

## Figures and Tables

**Table 1:** The main symbols and its meanings.

v	The value of a customer obtained from purchasing a traditional product
c	The distance cost when a customer buys a product
$p_{a}$	The price of traditional product a
$p_{b}$	The price of traditional product b
$p_{p}$	The price of probabilistic product
x	The heterogeneity of each consumer
$x_{a}$	The marginal consumer who obtains the same utility from purchasing traditional product a and the probabilistic product
$x_{b}$	The marginal consumer who obtains the same utility from purchasing traditional product b and the probabilistic product
β	Consumer’s risk aversion level for purchasing probabilistic product
φ	The probability of traditional product a becoming the probabilistic product
$w_{a}$	The wholesale price that the manufacturer A offers to a retailer for the traditional product
$w_{b}$	The wholesale price that the manufacturer B offer to a retailer for the traditional product
$w_{a}^{p}$	The wholesale price that the manufacturer A offer to a retailer for the probabilistic product
$w_{b}^{p}$	The wholesale price that the manufacturer B offer to a retailer for the probabilistic product
ρ	The relative bargaining power of a retailer

## Appendix A

1. The retailer’s best reply functions are as follows:

$$\begin{array}{c} p_{a}^{*}=\frac{-c+2v-4v\beta -3c\beta^{2}+2c\beta^{2}+4c\beta^{2}\varphi +\left(1+\beta \right)\left[-1+\beta \left(2\varphi -1\right)\right]p_{b}}{1+\beta \left(2\varphi -7\right)+\beta^{2}\left(4\varphi -1\right)+\beta^{3}\left(2\varphi -1\right)}\\ +\frac{\beta \left[-3-2\beta \varphi \left(\rho -1\right)+2\rho \varphi +\beta^{2}\left(2\varphi -1\right)\right]w_{a}}{1+\beta \left(2\varphi -7\right)+\beta^{2}\left(4\varphi -1\right)+\beta^{3}\left(2\varphi -1\right)}+\frac{\left(2\beta \varphi -2\beta^{2}\rho -2\beta \rho \varphi +2\beta^{2}\rho \varphi \right)w_{b}}{1+\beta \left(2\varphi -7\right)+\beta^{2}\left(4\varphi -1\right)+\beta^{3}\left(2\varphi -1\right)}\\ p_{p}^{*}=\frac{-2c+3v+c\beta -3v\beta +v\beta^{2}+c\beta^{3}-v\beta^{3}+c\beta \varphi -2v\beta \varphi -2c\beta^{2}\varphi -3c\beta^{3}\varphi +2v\beta^{3}\varphi +2c\beta^{2}\varphi^{2}+2c\beta^{3}\varphi^{2}}{1+\beta \left(2\varphi -7\right)+\beta^{2}\left(4\varphi -1\right)+\beta^{3}\left(2\varphi -1\right)}\\ \;+\frac{\left[\beta \left(4\varphi -2\right)-2\right]p_{b}+\left[-1+\rho \varphi +\beta^{2}\left(-1+2\varphi \right)\left(1+\rho \varphi \right)+2\beta \left(1-\varphi -2\rho \varphi +{\rho \varphi }^{2}\right)\right]w_{a}}{1+\beta \left(2\varphi -7\right)+\beta^{2}\left(4\varphi -1\right)+\beta^{3}\left(2\varphi -1\right)}\\ +\frac{\left(\rho -4\beta \rho -\beta^{2}\rho -\rho \varphi +6\beta \rho \varphi +3\beta^{2}\rho \varphi -2\beta \rho \varphi^{2}-2\beta^{2}\rho \varphi^{2}\right)w_{b}}{1+\beta \left(2\varphi -7\right)+\beta^{2}\left(4\varphi -1\right)+\beta^{3}\left(2\varphi -1\right)} \end{array}$$

2. The marginal profits of the two manufacturers are as follows:

$$\;\begin{array}{l} \frac{\partial \pi_{A}}{\partial w_{a}}=\frac{A_{1}+A_{2}p_{b}-A_{3}w_{a}+A_{4}w_{b}}{c\left[1+\beta \left(2\varphi -1\right)\right]\left[1+\beta \left(2\varphi -7\right)+\beta^{3}\left(2\varphi -1\right)+\beta^{2}\left(4\varphi -1\right)\right]}\\ \frac{\partial \pi_{B}}{\partial w_{b}}=\frac{A_{5}+A_{6}p_{b}+A_{7}w_{a}+(8\beta \rho -8\beta \rho \varphi )w_{b}}{c\left[1+\beta \left(2\varphi -1\right)\right]\left[1+\beta \left(2\varphi -7\right)+\beta^{3}\left(2\varphi -1\right)+\beta^{2}\left(4\varphi -1\right)\right]}\\ \frac{\partial \pi_{B}}{\partial p_{b}}=\frac{A_{8}-A_{9}p_{b}+A_{10}w_{a}+A_{11}w_{b}}{c\left[1+\beta \left(2\varphi -1\right)\right]\left[1+\beta \left(2\varphi -7\right)+\beta^{3}\left(2\varphi -1\right)+\beta^{2}\left(4\varphi -1\right)\right]} \end{array}$$

where

$A_{1}=c-v-3c\beta +v\beta +c\beta^{2}+v\beta^{2}+c\beta^{3}-v\beta^{3}+3c\beta \varphi -2v\beta \varphi -4c\beta^{2}\varphi -3c\beta^{3}\varphi +2v\beta^{3}\varphi -3c\beta \rho \varphi +4v\beta \rho \varphi +4c\beta^{2}\rho \varphi -4v\beta^{2}\rho \varphi -c\beta^{3}\rho \varphi +2c\beta^{2}\varphi^{2}+2c\beta^{3}\varphi^{2}-2c\beta^{2}\rho \varphi^{2}+2c\beta^{3}\rho \varphi^{2}$
$A_{2}=\left[1+\beta^{3}\rho \varphi \left(-1+2\varphi \right)+\beta \left(-2+2\varphi -3\rho \varphi \right)+\beta^{2}\left(1-2\varphi +2\rho \varphi^{2}\right)\right]$
$A_{3}=4\beta \left\{-1+\rho \varphi +\beta^{2}\rho \varphi \left(-1+2\varphi \right)+\beta \left[1-2\varphi -2\left(-1+\rho \right)\rho \varphi^{2}\right]\right\}$
$A_{4}=-\beta \rho +\beta^{3}\rho +\beta \rho \varphi -2\beta^{2}\rho \varphi -3\beta^{3}\rho \varphi +4\beta^{2}\rho^{2}\varphi +2\beta^{2}\rho \varphi^{2}+2\beta^{3}\rho \varphi^{2}-4\beta^{2}\rho^{2}\varphi^{2}$
$A_{5}=\beta \rho \left(\varphi -1\right)\left(-3c+4v+4c\beta -4v\beta -c\beta^{2}-2c\beta \varphi +2c\beta^{2}\varphi \right)$
$A_{6}=2\beta \rho \left(\varphi -1\right)\left(1+\beta \right)\left[-1+\beta \left(-1+2\varphi \right)\right]$
$A_{7}=\beta \rho \left(\varphi -1\right)\left(-1+\beta^{2}-2\beta \varphi -2\beta^{2}\varphi +4\beta \rho \varphi \right)$
$A_{8}=v-2c\beta -5v\beta +c\beta^{2}+3v\beta^{2}+v\beta^{3}+c\beta^{4}+c\beta \varphi +2v\beta \varphi -6c\beta^{2}\varphi -3c\beta^{3}\varphi -2v\beta^{3}\varphi -4c\beta^{4}\varphi +2c\beta^{2}\varphi^{2}+6c\beta^{3}\varphi^{2}+4c\beta^{4}\varphi^{2}$
$A_{9}=2\left[1+\beta^{2}+\beta \left(-5+2\varphi \right)+\beta^{3}\left(-1+2\varphi \right)\right]$
$A_{10}=\beta \left[-1+\rho \varphi +\beta^{2}\left(-1+2\varphi \right)\left(1+\rho \varphi \right)+2\beta \left(1-\varphi -2\rho \varphi +\rho \varphi^{2}\right)\right]$
$A_{11}=-2\beta \rho -4\beta^{2}\rho -2\beta^{3}\rho +2\beta \rho \varphi +8\beta^{2}\rho \varphi +6\beta^{3}\rho \varphi -4\beta^{2}\rho \varphi^{2}-4\beta^{3}\rho \varphi^{2}$
